# Different Myosin Head Conformations in Bony Fish Muscles Put into Rigor at Different Sarcomere Lengths

**DOI:** 10.3390/ijms19072091

**Published:** 2018-07-18

**Authors:** Felicity Eakins, Jeffrey J. Harford, Carlo Knupp, Manfred Roessle, John M. Squire

**Affiliations:** 1 School of Medicine, Imperial College, London SW7 2AZ, UK; felicity.eakins@gmail.com (F.E.); Jeff_harford@yahoo.co.uk (J.J.H.); c.knupp@cardiff.ac.uk (C.K.); 2 School of Optometry and Vision Science, Cardiff University, Cardiff CF19 3NB, UK; 3 European Synchrotron Radiation Facility, CS 40220, 38043 Grenoble, France; manfred.roessle@fh-luebeck.de; 4 School of Physiology, Pharmacology and Neuroscience, University of Bristol, Bristol BS8 1TD, UK

**Keywords:** rigor muscle, myosin cross-bridge cycle, steric blocking mechanism, roll and lock mechanism, low-angle X-ray diffraction, synchrotron radiation, bony fish muscle

## Abstract

At a resting sarcomere length of approximately 2.2 µm bony fish muscles put into rigor in the presence of BDM (2,3-butanedione monoxime) to reduce rigor tension generation show the normal arrangement of myosin head interactions with actin filaments as monitored by low-angle X-ray diffraction. However, if the muscles are put into rigor using the same protocol but stretched to 2.5 µm sarcomere length, a markedly different structure is observed. The X-ray diffraction pattern is not just a weaker version of the pattern at full overlap, as might be expected, but it is quite different. It is compatible with the actin-attached myosin heads being in a different conformation on actin, with the average centre of cross-bridge mass at a higher radius than in normal rigor and the myosin lever arms conforming less to the actin filament geometry, probably pointing back to their origins on their parent myosin filaments. The possible nature of this new rigor cross-bridge conformation is discussed in terms of other well-known states such as the weak binding state and the ‘roll and lock’ mechanism; we speculate that we may have trapped most myosin heads in an early attached strong actin-binding state in the cross-bridge cycle on actin.

## 1. Introduction

**Figure 1:**
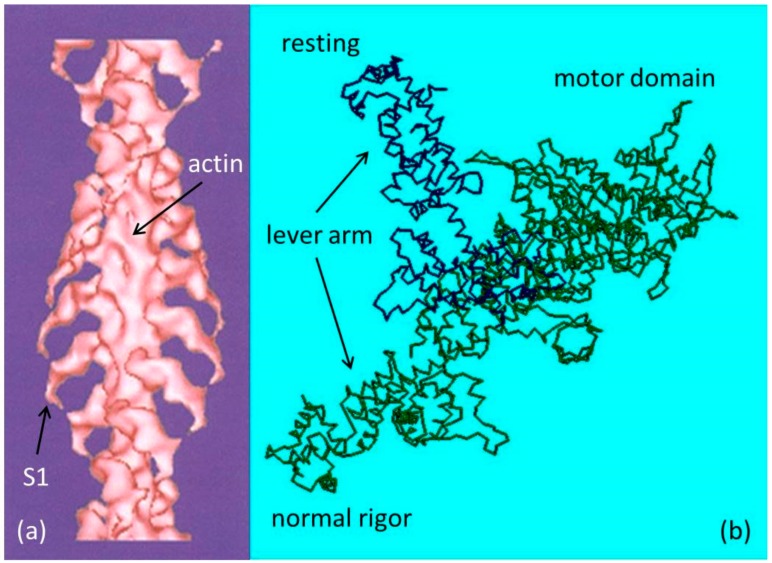
(**a**) Electron density map (low resolution) of F-actin labelled with myosin S1 fragments, with no ATP present based on electron microscopy and 3D reconstruction; (**b**) Representation of the myosin head with the lever arm up as in relaxed muscle and down as in normal rigor (**a**), assuming a vertical actin filament on the right of the motor domain.

Rigor is the state into which a muscle enters in the absence of ATP (Adenosine triphosphate). In relaxed muscle there is always a population of myosin heads associated with actin. In the presence of ATP and absence of calcium these associations are weak, very short lived, and produce no force. When ATP is removed from a muscle cell the dissociation of actin and myosin, which requires ATP, cannot take place. The two proteins become trapped in an attached state. The myosin heads, projecting from the thick filaments, form permanent strong bonds with the actin monomers in the thin filaments. These strongly attached states of myosin heads on actin monomers in the absence of ATP are known as rigor complexes and are thought to be similar in configuration to the AM step in the cross-bridge cycle in contracting muscle [1]. These strong rigor attachments cause the muscle to become stiff as in rigor mortis. Knowledge of the structure of the rigor complex has come principally from electron microscopy (EM) reconstructions of isolated thin filaments decorated with isolated myosin heads (myosin subfragment-1 or S1). The atomic arrangement in S1 has been determined by protein crystallography Figure 1b [2]. Crystallography can only so far give the separate structures of actin and myosin molecules, as they have proved difficult to crystallise whilst bound to each other. The crystal structures can, however, be docked into the EM reconstructions allowing higher resolution structures of the complex to be obtained [3,4]. A low resolution structure of this type (Figure 1a) shows the tilted configuration of all the myosin heads on the thin filament. Higher resolution structures have been determined by Holmes et al. [4] and von der Ecken et al. [5].

These reconstructions only give information about how isolated myosin heads interact with actin. It is likely that within rigor muscle, where both heads of each myosin molecule bind to actin and they are tethered back to their parent myosin filaments, the conformation of the two heads will differ not only from each other and the isolated S1 conformation, but even from the conformation of heads in other myosin molecules within the filament [6]. This is due to the different periodicities of the thick and thin filament and the steric constraints created because the two heads of each myosin molecule must bind to different actin monomers [7]. EM structures of the rigor complex within whole muscle have been produced from tomograms of insect flight muscle (IFM) in rigor [6]. Models were built to fit class averages of 11 different conformations of myosin S1 bound to actin. However, the periodicities of the thick and thin filaments in IFM are different from those in vertebrate muscle, suggesting that the conformations of the rigor complexes may also differ. 

Modelling has also been carried out using X-ray diffraction patterns from rigor vertebrate muscles. Squire and Harford [8] used the idea of actin target zones [9,10] where cross-bridges can more easily bind to the thin filament due to the more favourable actin monomer orientation or the position of the thin filament regulatory proteins. Linking this to parameters governing the extent of movement which the myosin head can perform, Squire and Harford [8] calculated possible labelling patterns and the resulting diffraction patterns and compared them to the experimental X-ray data. The modelling only successfully reproduced the rigor layer-lines when actin target areas were included, providing good evidence for the concept. Crystallographic analysis has also been used to explain the layer-line pattern in X-ray diffraction patterns from rigor muscle [11]. This analysis identified rigor layer-lines originating from myosin heads stereospecifically bound to actin, but still showing the effect of their repeat on the thick filament. These rigor layer-lines could give useful information about the actin and myosin interaction in contracting muscle. More recently, Koubassova and Tsaturyan [12] used a different method to model X-ray layer-line intensities from rabbit muscle fibres in rigor. Using a concept they called the principle of ’minimal elastic distortion energy’, labelling patterns were created of myosin heads on the actin filaments within a unit cell. The intensities of the layer-lines were then calculated using high resolution structures of actin, S1, and the actomyosin complex, which were allowed to bend at certain points to follow the labelling pattern. Lattice disorder was also built into the calculations. This process allowed a possible model of the rigor unit cell to be found. Contributions from the myosin backbone, myosin subfragmant-2 (S2), and the thin filament regulatory proteins were not taken into account in the calculation of the layer-line intensities and the match between experimental data and the model was judged by eye. This demonstrates, however, the possibility of setting up this protocol to model the rigor X-ray diffraction pattern to obtain a structure for the rigor unit cell.

Analysis of the equator from the low angle X-ray diffraction pattern of rigor muscle gives some of the clearest data about the attachment of myosin cross-bridges to actin. The distribution of mass within the unit cell as viewed down the filament axis can be calculated by producing electron density maps from the equatorial reflections, using the process of Fourier synthesis. This process has already been carried out for resting, active, and short sarcomere length rigor in bony fish muscle and the results compared [13]. 

Conventionally, when muscles are put into rigor, they are kept at very low tension to prevent damage as they enter rigor. This is because, during the process of entering rigor, shortening may occur within the muscle. This builds up tension and can damage the muscle if it is held taut. Therefore, most studies have looked at changing the sarcomere length (SL) of a muscle after it has been put into rigor at a short SL. For example, X-ray diffraction patterns were taken from single rigor frog muscle fibres whilst undergoing a stretch and the interference fine structure of the M3 reflection was followed to give information about the tilting of the myosin heads occurring as a result of the stretch [7]. However, the length changes involved here were rather small (about 150 nm per half sarcomere). Also tomograms of stretched IFM in rigor have been taken and modelled to give information about the angles of the distorted cross-bridges [14]. Little work has been carried out on muscle put into rigor after first being stretched to longer sarcomere lengths. 

In the present study, whole bony fish fin muscles were put into rigor at two different sarcomere lengths. Some muscles were set to a sarcomere length of 2.2 µm. This gives full overlap of the actin and myosin filaments and is the typical length used for rigor preparations. Other muscles were set to a longer SL of 2.5 µm and then, using the same protocol as for the shorter muscles, put into rigor. Two studies were involved. In one, turbot fin muscles at both sarcomere lengths were studied on a laboratory X-ray source and, in the second, plaice fin muscles at both lengths were studied using ESRF synchrotron radiation. BDM (2,3-butanedione monoxime) was used in the rigor solutions because of its ability to prevent muscles developing rigor force, hence reducing damage to the muscles as they enter rigor, especially at the longer sarcomere length (see Squire and Knupp, [15] and references therein). In the first study, intact whole muscles were also prepared and kept in Ringer solution at SL = 2.45 µm, to allow comparison of the rigor structures with the relaxed muscle state. X-ray diffraction patterns were recorded from all preparations to obtain information about the rigor myosin cross-bridge conformations at different initial SLs. Unexpectedly, the patterns from the longer length muscles were not just weaker versions of the patterns from muscles at full overlap; they were quite distinct and very interesting.

## 2. Results 

The X-ray diffraction pattern from rigor muscle at short sarcomere length has been extensively studied. The pattern consists of a set of layer-lines and meridionals which can be indexed on a repeat of approximately 2145 Å, which is 5 × 429 (the myosin filament repeat) and 3 × 715 Å (the actin filament pitch) [15]. Therefore, layer-lines are observed at some of the positions of the actin and myosin layer-lines in relaxed muscle (although with different intensity distributions), but also at positions in between these, dependent on the beat period between actin and myosin: 2145 Å. The layer-lines which only occur in patterns from rigor muscle are therefore referred to as beating layer-lines [12]. The first few of these beating layer-lines were identified by Haselgrove [16] and since then they have been observed at several other spacings, reported by Squire and Harford [8]; Yagi [11]; Koubassova and Tsaturyan [12]; and references therein. The equator of the pattern consists of the same set of reflections seen in relaxed muscle patterns and originating from the hexagonal and tetragonal lattices of the A-band and Z-line, respectively. However, the relative intensities of the reflections differ in rigor compared to relaxed muscle. In particular, the ratio of the A(10) and A(11) intensities (A referring to A-band reflections rather than Z-band reflections) reduces by a large extent from the relaxed to the rigor state, consistent with myosin head mass moving from around myosin in resting muscle to around actin in active and rigor muscles. 

### 2.1. Results from Laboratory-Based Experiments

**Figure 2:**
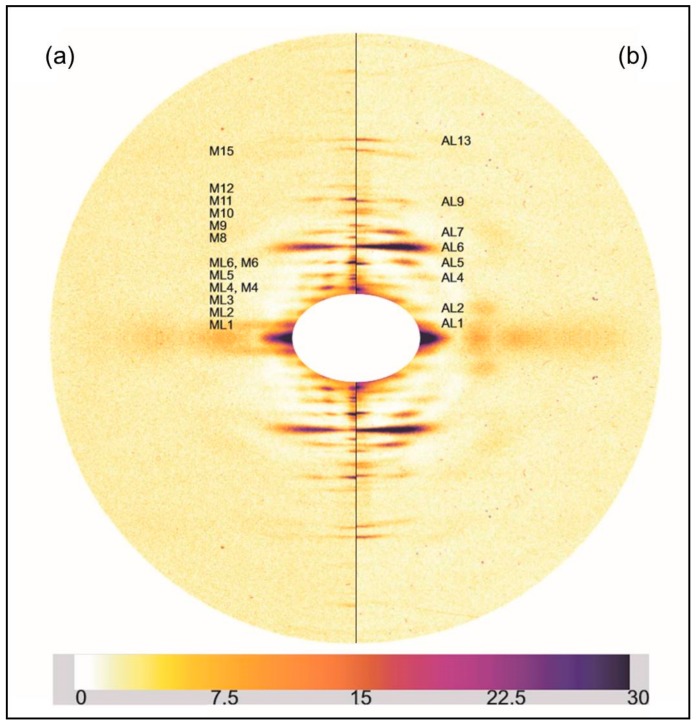
(**a**) Left half of the X-ray diffraction pattern from relaxed Turbot fin muscle, 8 h exposure time. Myosin layer-lines (orders of 429.6 Å) are labelled ML1 to ML6 and meridionals M4 to M15; (**b**) Right half of the X-ray diffraction pattern from the short SL rigor Turbot fin muscle, 8 h exposure time. Actin layer-lines are labelled AL1 to AL13. The meridian is vertical, the equator horizontal. The lower strip shows the intensity scale used.

Initially the two rigor diffraction patterns were compared to the pattern from the relaxed state. Figure 2 shows the left half of the relaxed X-ray pattern and the right half of the short SL rigor pattern from Turbot fin muscle. The myosin layer-lines (ML), meridional reflections (M), and actin layer-lines (AL) are labelled. The short SL rigor pattern shows a number of differences to the relaxed pattern. As seen in previous studies, the myosin layer-lines largely disappear and the actin layer-lines become more intense. The reciprocal spacings of the myosin meridional reflections become slightly smaller, showing a roughly 1% increase in the myosin filament periodicity in rigor muscle (detailed later). Some of the myosin meridional reflections disappear, in particular the M5, M8, and M12 reflections are not visible in the rigor pattern and the relative intensities of the other meridional reflections change. The M4 and M15 reflections increase in intensity, while the M6 and M11 reflections weaken. 

**Figure 3:**
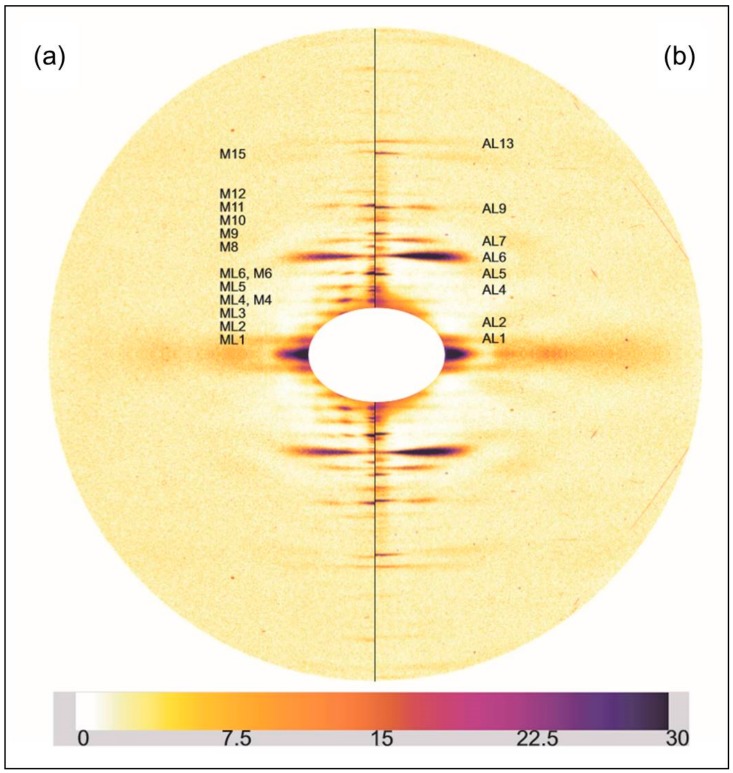
(**a**) Left half of the X-ray diffraction pattern from relaxed Turbot fin muscle, 8 h exposure time. Myosin layer-lines (orders of 429.6 Å) are labelled ML1 to ML6 and meridionals M4 to M15; (**b**) Right half of the X-ray diffraction pattern from the longer (2.5 µm) SL rigor Turbot fin muscle, 8 h exposure time. Actin layer-lines are labelled AL1 to AL13. The meridian is vertical, the equator horizontal. The intensity scale is shown at the bottom.

Figure 3 shows a comparison similar to Figure 2, but this time between relaxed and the longer sarcomere length (2.5 µm) rigor diffraction patterns. Again actin and myosin layer-lines and meridionals are labelled. The changes in the longer SL rigor pattern are broadly similar to those seen in the short SL rigor. The myosin layer-lines reduce and the actin layer-lines become more intense. The myosin meridional reflections have a larger d-spacing and the intensities of the myosin meridional reflections change. 

**Figure 4:**
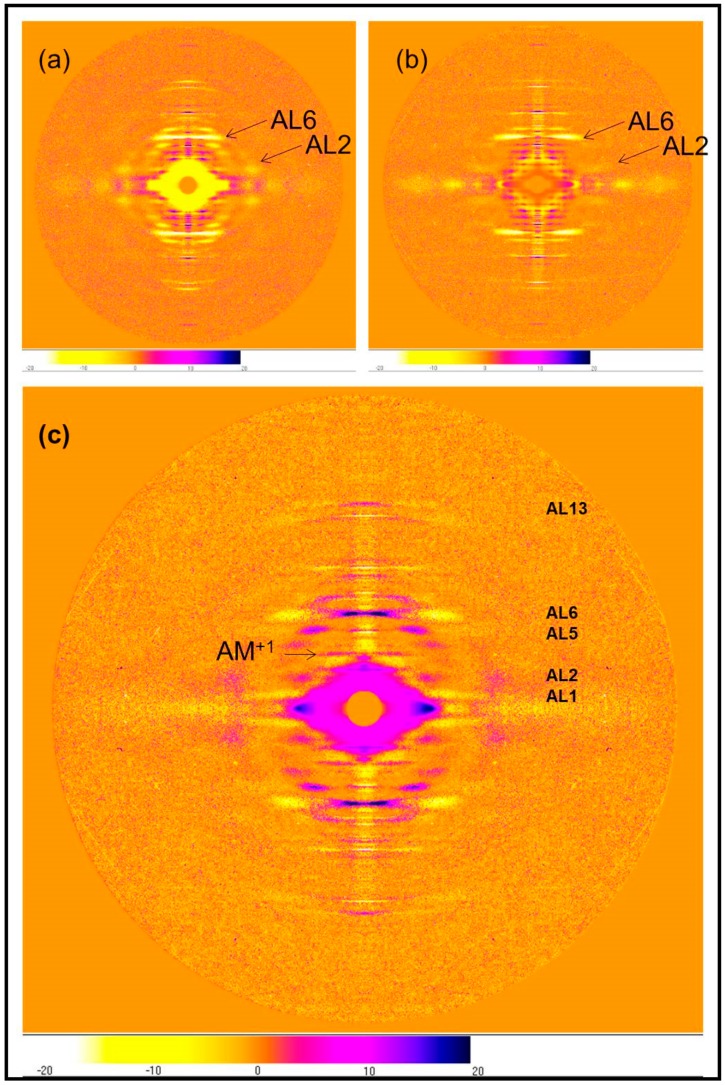
(**a**) The difference pattern relaxed minus 2.2 µm rigor; (**b**) The difference pattern relaxed minus 2.5 µm rigor; (**c**) The difference pattern of the short SL rigor pattern minus the longer SL rigor pattern. All from Turbot fin muscle, 8 h exposure. Actin layer-lines are labelled and the beating actin myosin layer-line is labelled AM^+1^ (Koubassova and Tsaturyan [12]). The positive and negative differences are coded as in the lower strip for all patterns; reds, purple, and black show increases, light orange and yellows show decreases, dark orange shows no change.

The differences between the two rigor patterns are more clearly illustrated by producing difference maps for the two rigor patterns with the relaxed pattern and with each other. Figure 4 shows the difference maps obtained by subtracting the two rigor patterns from the X-ray diffraction pattern of relaxed Turbot fin muscle. The left pattern Figure 4a is the difference between relaxed and the short SL rigor and the right pattern, Figure 4b is the difference between the relaxed state and the longer SL rigor. The lower figure (c) shows the long SL pattern subtracted from the short SL pattern. The differences in the actin layer-line intensities can be seen clearly; the short rigor has enhanced actin layer lines at a smaller reciprocal radius than relaxed muscle, whereas the longer rigor pattern only has greatly increased intensity on AL6, with a smaller increase on AL7. Compared with relaxed, in the longer SL rigor pattern AL2 is virtually absent at the arrowed position in Figure 4b, whereas AL2 increases greatly in the short SL rigor pattern at that position (Figure 4a). 

**Figure 5:**
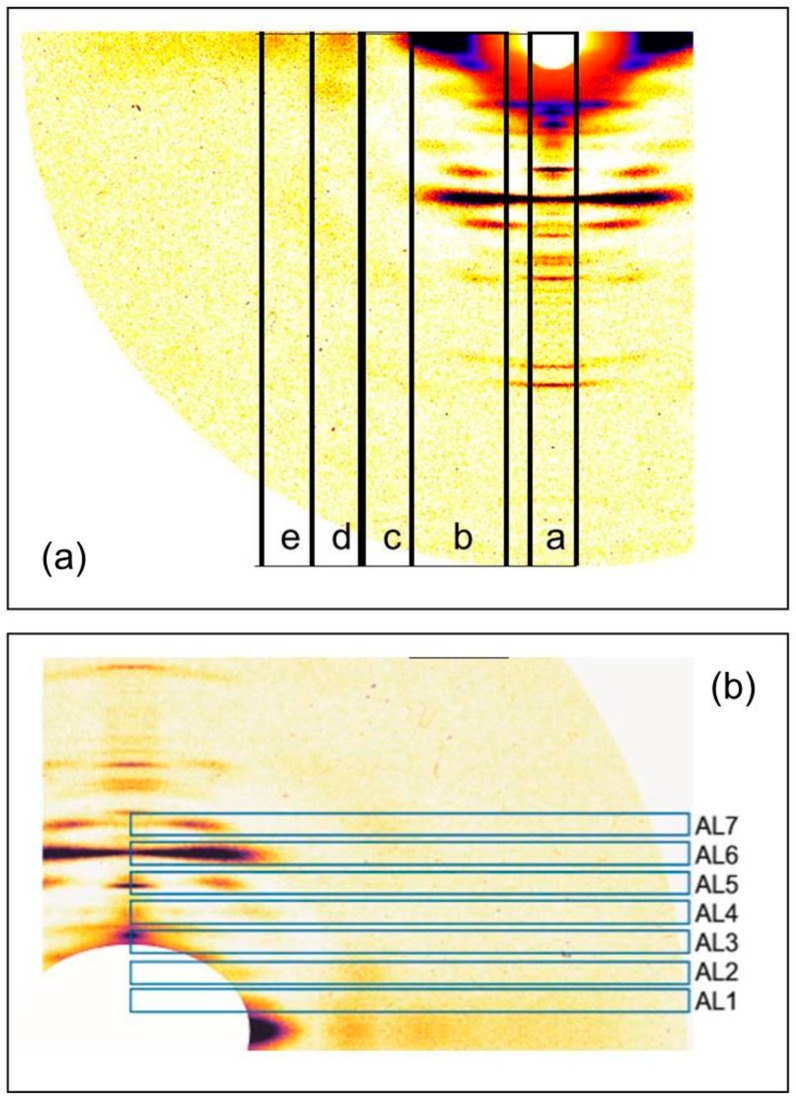
(**a**) Positions and widths of the horizontally integrated profiles taken parallel to the meridian of the X-ray patterns from the three muscle states: relaxed, short SL rigor, and longer SL rigor; (**b**) Positions and widths of the vertically integrated profiles taken along the actin layer-lines in the X-ray diffraction patterns from the three muscle states. The traces along these regions are shown in Figure 6 and Figure 7.

**Figure 6:**
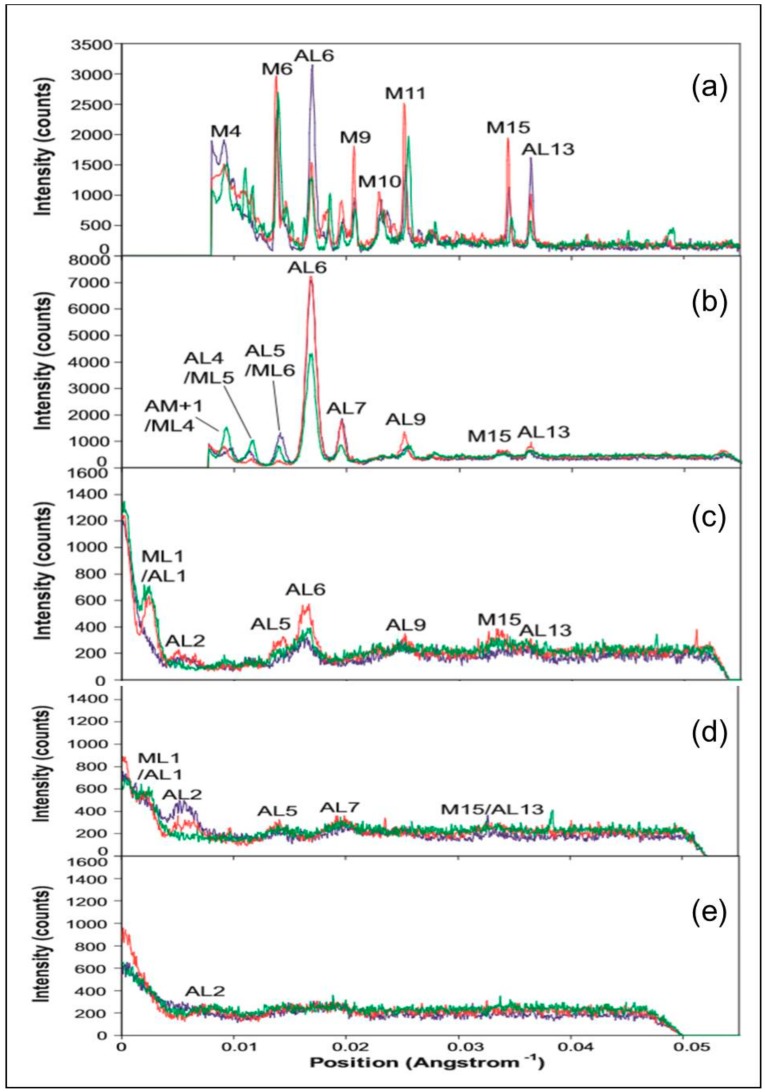
Integrated intensity profiles in the strips indicated in Figure 5a, which are all parallel to the meridian of the diffractions patterns. Layer lines referred to in the text are labelled. Trace colours: relaxed state (green), short rigor (blue), and longer rigor (red). (**a**) Meridional profile integrated out to 0.002123 Å^−1^ on either side of the meridian; (**b**) Profile parallel to the meridian (axial profile) centred on the peak of the 6th actin layer-line horizontally integrated from 0.003663 to 0.01428 Å^−1^; (**c**) Axial profile horizontally integrated from 0.01428 to 0.01959 Å^−1^; (**d**) Axial profile horizontally integrated from 0.01959 to 0.02490 Å^−1^; (**e**) Axial profile horizontally integrated from 0.02490 to 0.03021 Å^−1^.

The differences in intensity and peak position between the three muscle states can be more easily quantified by looking at integrated profiles along the row-lines and layer-lines of interest. Horizontally integrated profiles were therefore taken along lines parallel to the meridian of the X-ray patterns from the three muscle states, at five positions as shown in Figure 5a. The integration strips were along the meridian of the X-ray patterns, at the peak of the 6th actin layer-line, and at three further positions of consecutively higher radii. The profiles are shown in Figure 6. Figure 6a, along the meridian of the patterns, shows the consistently smaller reciprocal spacing of the myosin meridional peaks from relaxed to the short and long SL rigors. The coincidence of the actin peaks, which do not appear to change their spacings between the three muscle states, can also be seen. 

There is significant variation in the intensity of each particular reflection between muscle states. In particular, across the meridian (Figure 6a), the AL6 is approximately twice as strong in the short SL rigor compared to the relaxed and longer SL rigor states which have similar intensities. In the case of the profile at the position of the AL6 peak, Figure 6b shows interesting differences between the X-ray patterns from the three different muscle states. The total off-meridional intensity of the 6th actin layer-line within this profile is very similar for both rigor states but is approximately twice that in relaxed muscle. However, for both AL9 and AL13 the intensity is greater in the longer SL rigor with the relaxed and short SL rigor intensities being similar. This profile also clearly shows the very low intensity on AL4 and AL5 in the longer SL rigor compared to the short SL rigor at this radius. The beating actin-myosin layer-line AM^+1^ in this profile appears only to be visible in the short SL rigor. Interestingly, the peak in this area in the longer SL rigor appears to be at the position of ML4 rather than the beating layer-line. 

At the position of the profile in Figure 6c there is more intensity on AL5, AL6, AL9, and M15 in the longer SL rigor than in the relaxed and short SL rigor states. At the ML1/AL1 peak the longer SL rigor and relaxed states show similar intensities, but the short SL rigor shows none. In profile 6d all three patterns are very similar except at the 2nd actin layer-line where the short SL rigor has approximately twice as much intensity as the longer SL rigor and the relaxed pattern has no peak at this position. The profile in Figure 6e is similar to profile 6d except that at the AL2 position it is the longer SL rigor and the relaxed state which have more prominent peaks and the short SL rigor which has less.

**Figure 7:**
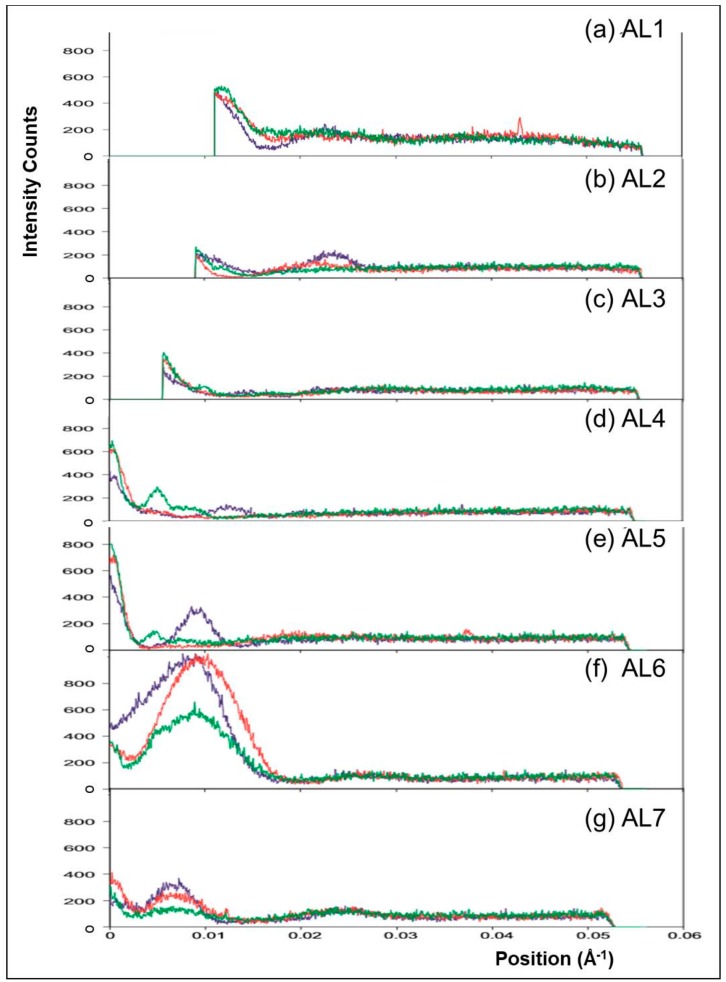
Vertically integrated horizontal profiles taken from the X-ray diffraction patterns from Turbot fin muscle in the relaxed state (green) and put into rigor at short (blue) and longer SL (red). The profiles are 0.00210 Å^−1^ wide and are centred on actin layer-lines 1 to 7 (**a**–**g**) as indicated.

In diffraction patterns from rigor muscles the actin layer-lines increase in intensity, compared to the relaxed state, due to stereospecific labelling of the actin filaments by myosin heads. The intensity distributions along the layer-lines depend upon the pattern of cross-bridge labelling. To compare the positions and relative intensities of the peaks on these layer-lines in the two rigor states, vertically integrated profiles were taken along AL1 to AL7 in the X-ray patterns from the three muscle states. Figure 5b shows the positions and widths of these integrated profiles. Figure 7a–g show plots of the profiles along each layer-line in the three muscle states. Figure 7a shows the profile along AL1. Both the relaxed and the longer SL rigor patterns show a similar intensity profile with a large peak closest to the meridian overlapping a weaker peak at higher radius. The short SL rigor pattern also shows these peaks, but the width of the higher radius peak is smaller for this state and so does not overlap as much with the low radius peak as in the other two states.

Figure 7b shows the profile along AL2. The relaxed pattern shows no peaks along this layer-line, but both the rigor states have a peak at a similar position to the higher radius peak on AL1. However, the centre of this peak is closer to the meridian in the longer SL rigor pattern, the width is larger and the maximum intensity lower.

Actin layer-line three (AL3) in Figure 7c, shows no particularly strong peaks in any of the three muscle states. Apart from at the meridian, AL4 in Figure 7d shows weak peaks at low radius, but at different radial positions in the two rigors. Figure 7e shows the AL5 profiles for the three muscle states. The absence of peaks on this layer-line in the longer SL rigor pattern can be seen, except for intensity on the meridian where the M6 reflection overlaps with this layer-line. The short SL rigor profile, in contrast, shows a large peak (other than the M6) which is closer to the meridian than the peak in AL4 and is roughly at the same radius as the peak on AL6 (see below). The relaxed profile has some small peaks close to the meridian, but again, these are from the partial overlap of ML6 with this layer-line. 

Actin layer-line six (AL6) contains the most intense peak out of all the actin layer-lines and this profile is displayed in Figure 7f. All three muscle states show an intense peak close to the meridian, although the relaxed peak is about half as intense as that in the two rigor patterns. Both the rigor patterns show a similar maximum peak height and all three states have a maximum at very similar radial positions. However, the shape of the short SL rigor peak is different to that in the other two states, being quite asymmetric with a great deal of intensity towards the meridian. All three patterns also show a weak peak at a higher radius. The longer SL rigor and relaxed profile both show a small meridional peak which is not apparent as a separate peak in the short SL rigor. Finally, Figure 7g shows the profiles of AL7 in the three muscle states. All three show two peaks other than the central meridional, which is most likely from a slight overlap with the M9 reflection. The higher radius peak (at around 0.025 Å^−1^) is very similar in shape and intensity for all three patterns. However, the lower radius peak (at around 0.007 Å^−1^) has different intensities for the three muscle states, with relaxed being the weakest and the short SL rigor the strongest.

### 2.2. Results from Synchrotron Experiments

**Figure 8:**
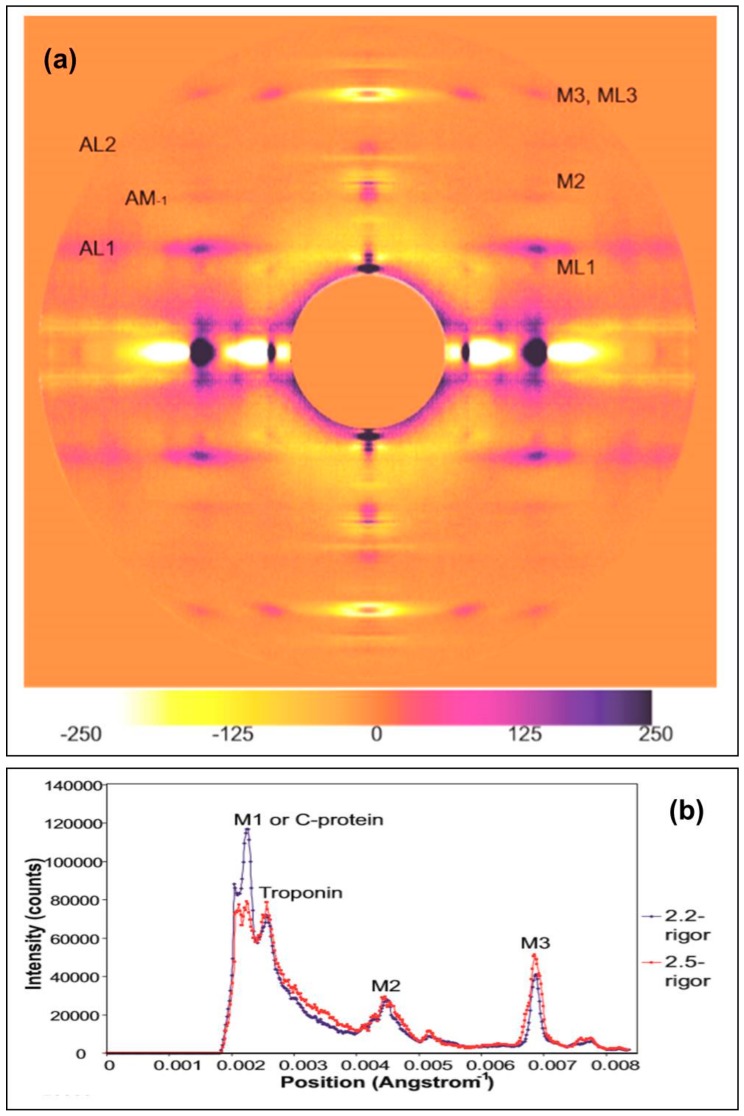
(**a**) Difference map created by subtracting the synchrotron X-ray diffraction pattern of Plaice fin muscle put into rigor at SL = 2.5 µm from the diffraction pattern of Plaice fin muscle put into rigor at SL = 2.2 µm. The actin and myosin layer-lines are labelled AL1 to AL2 and ML1 to ML3 respectively, the myosin meridionals M2 to M3 and the beating actin myosin layer line AM^−1^. An attenuation strip was used to reduce the total intensity of the equatorials; (**b**) Horizontally integrated profiles of the meridian in the two summed X-ray diffraction patterns from Plaice fin muscle put into rigor at two different sarcomere lengths 2.2 µm (blue trace) and 2.5 µm (red trace). The meridional reflections are labelled M1 to M3. The fine sampling on the meridian will change with sarcomere length due to interference between the two halves of the A-band [7].

Similar experiments to those described above were carried out on the ID2 bypass beamline at the ESRF with a longer effective camera length so that the central part of the patterns in Figure 2 and Figure 3 could be recorded. The muscles used were from Plaice rather than Turbot. Figure 8a shows a difference intensity map between patterns from rigor Plaice fin muscles put into rigor at SL = 2.2 and 2.5 µm respectively. The comparison reveals differences between the two low-angle rigor diffraction patterns. The first actin layer-line is stronger in the shorter SL rigor, in particular close to the meridian at the (1,1) row-line position. However, both patterns show little intensity along the inner end of AL2. Remnants of the first myosin layer-line are visible in both, at the (1,0) row line position. However, this reflection is wider in the horizontal direction in the longer SL rigor. The M2 reflection is wider along the meridian in the 2.2-rigor, while the M3 is wider along the meridian in the 2.5-rigor. The M3 reflection is also wider horizontally in the longer SL rigor. The third myosin layer-line shows intensity at both the (1,0) and (1,1) row line positions in both of the X-ray patterns and in the long SL rigor these reflections appear to be more smeared along the layer-line than in the short SL rigor. The short SL rigor shows some weak intensity along what appears to be the beating actin myosin layer-line, AM^−1^, at 240 Å^−1^, particularly at the (1,1) row line position as for AL1, but very little intensity can be seen at this position in the longer SL rigor.

#### 2.2.1. Differences on the Equator

**Figure 9:**
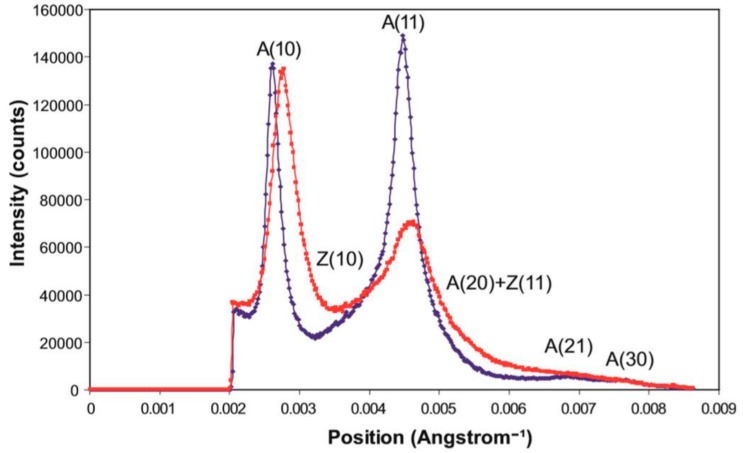
Equatorial profiles from the summed X-ray diffraction patterns of Plaice fin muscle put into rigor at SL = 2.2 µm (blue trace) and 2.5 µm (red trace), integrated from 0 to 0.00043 Å^−1^ on either side of the equator. The positions of the six equatorial reflections (including Z-line peaks) present in these profiles are labelled.

On the equator (Figure 9) it can be seen that the reflections in the longer SL rigor are slightly further from the pattern centre, indicating a smaller unit cell in the longer SL rigor. Both the (1,0) and (1,1) reflections are also wider in the longer SL rigor, showing less lateral order than in full overlap rigor. However, the longer SL rigor has more intensity in the higher angle equatorial peaks. Peak fitting of the first few equatorial peaks gave the relative intensities listed in Table 1. 

A ratio which is often used as a measure of the number of myosin heads attached to actin is the intensity ratio of the A(10) and A(11) reflections; the implication is that the stronger the A(11) is, the more heads are likely to be bound to actin. This is a blunt instrument in that other factors also define these relative intensities, such as the shape of the myosin heads on actin, but for the record the two rigors give A(10)/A(11) intensity ratios of 0.42 (i.e., A(10) < A(11)) for the short rigor and 0.99 (i.e., A(10) = A(11)) for the longer rigor. 

#### 2.2.2. Changes on the Meridian

The strong intensity of the M3 peak in Figure 8b from the longer sarcomere length rigor clearly shows the splitting of this reflection into three closely-spaced peaks along the meridian due to interference between the two halves of the A-band. The position of the M3 peak in the two rigors (*P*_average_ Å) was estimated, relative to the resting M3 spacing of 143.2 Å determined by Harford and Squire [17], using the weighted mean formula. For example for three closely spaced sub-peaks:

*P*_average_ = (*I*_peak1_*P*_peak1_ + *I*_peak2_*P*_peak2_ + *I*_peak3_*P*_peak3_)/(*I*_peak1_ + *I*_peak2_ + *I*_peak3_) (1)

where *P* is position and *I* is intensity. The mean values (9 measurements) were *P*_average_ = 145.49 ± 0.24 Å for the 2.2 µm rigor and 145.89 ± 0.07 Å for the 2.5 µm rigor. These were not significantly different (*p* = 0.13) giving an average for the two rigors of 145.7 ± 0.2 Å. Compared with the resting value of 143.2 Å this is a 1.54% increase in M3 spacing from resting to rigor. 

The intensities of several meridional peaks were determined using FibreFix [18], and Peakfit (Table 2) and the intensities were corrected for the effect of their width across the meridian by multiplying the integrated intensities of the peaks by the full lateral width at half maximum (FWHM) of the peaks (Huxley et al. [19]). Their correction was originally put forward as being an approximation, but, in Appendix A here, we show that the correction is totally justified within limits. In summary, Table 2 shows that the M1/C-protein peak from the short rigor muscle is over three times more than that from the longer rigor. On the other hand the Troponin (385 Å) peak and the M2 and M3 peaks are all much stronger, often by a factor of 2, in patterns from the long sarcomere length rigor than the short rigor.

## 3. Discussion

### 3.1. Head Mass Attached to Actin

One of the first requirements in analysing the nature of the cross-bridge interactions with actin in the two different rigor states is to assess whether the average number of heads attached to actin per unit length of the thin filament is the same in the two cases. This is most easily done using X-ray diffraction data by calculating electron density maps of the fish muscle unit cell in projection down the fibre axis for the two muscle states using the observed equatorial reflections. This is achieved by Fourier synthesis [20] using the equation:

*ρ*(x,y) = Σ(±)F(h,k,0)[cos(2π(hx + ky))]*T*(h,k,0) (2)

*T*(h,k,0) is a standard temperature factor exp (−Bsin^2^θ/λ^2^) with *B* = 20,000 which is included to reduce series termination errors [20]. What are required are the relative amplitudes (*F*(h,k,0)) and the phases of the first few (we are using five) equatorial reflections in each case. The amplitudes can be measured directly (they are the square root of the intensities as in Table 1). However, the phases cannot easily be determined directly, but by various arguments two alternative sets of phases have been used in the past. Because the central few equatorial reflections represent structural information at low resolution, and at low resolution the muscle unit cell structure is approximately centrosymmetric (i.e., for every density at +x, +y in the unit cell there is an equivalent density at −x, −y), the phases instead of being anything between 0° and 360° can only be 0° or 180°.

**Figure 10:**
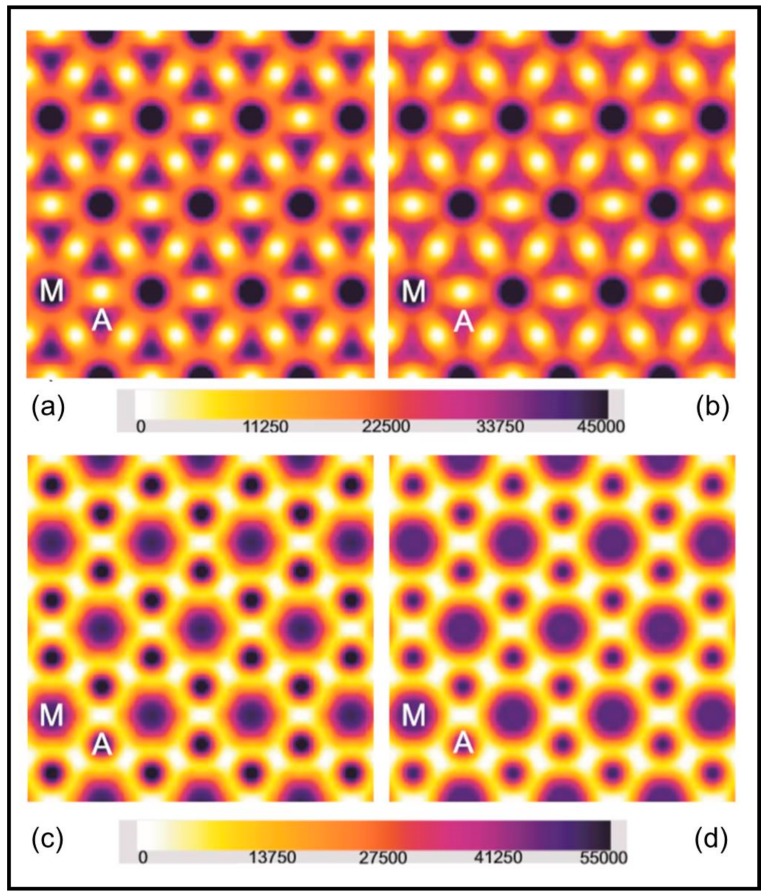
Electron density maps produced using the average amplitudes from the first five equatorial A-band reflections from Plaice fin muscle put into rigor at SL = 2.2 µm (**a**,**c**) and 2.5 µm (**b**,**d**); (**a**,**b**) were computed using phase set 1 (0°, 0°, 180°, 0°, 0°) and (**c**,**d**) were computed using phase set 2 (0°, 0°, 180°, 180°, 0°). A few unit cells of the A-band lattice are shown in each map and the actin and myosin filament positions are labelled A and M respectively. The colour scale used throughout is shown at the bottom. Densities were scaled to give the same total mass in each case.

The two preferred phase sets are phase set 1 (0°, 0°, 180°, 0°, 0°); [13,21,22,23] and phase set 2 (0°, 0°, 180°, 180°, 0°) [24]. Since it is still uncertain which set is correct, we have used both sets, but fortunately the results we need are approximately the same from both. The results are shown in Figure 10 for both rigor samples using both phase sets. The electron density maps were produced using the program Synthseries, updated and adapted for MS Windows by Carlo Knupp, December 2005, from the program FSYNTH, written by John Squire, February 1993, and updated by John Barry, September 1995.

Using the two phase sets it is clear that the electron density maps for the two rigor structures are rather different. There is much more mass at the actin positions in the short, full overlap, rigor (SL = 2.2 µm: Figure 10a,c) than in the longer rigor (SL = 2.5 µm; Figure 10b,d). One might expect this since the overlap of the myosin and actin filaments is less at the longer sarcomere length. But is there a population difference as well? In order to assess this we calculated the amount of electron density within a 90 Å radius of the centre of the actin filaments in the two cases (see [23,25]). For phase set 1 the relative masses were 1.163 × 10^7^ ± 0.016 × 10^7^ for the 2.2 µm rigor and 1.031 × 10^7^ ± 0.031 × 10^7^ for the longer rigor. For phase set 2 the masses were 1.167 × 10^7^ ± 0.016 × 10^7^ and 0.915 × 10^7^ ± 0.028 × 10^7^, respectively. The important number from these is their ratio. The ratio of the mass at 2.2 µm to that at 2.5 µm within the 90 Å radius for set 1 was 1.128 and for set 2 was 1.275. These can be compared with the ratio that would be obtained from knowledge of the sarcomere length if the labelling per unit actin filament length remains the same. In a half myosin filament in relaxed muscle there are 50 of the 143.2 spaced crowns (one level is missing but that does not significantly affect this calculation). The bridge region is therefore 49 × 143.2 = 7016.8 Å long in resting muscle. With the increased spacing in rigor this goes up to 49 × 145.7 = 7139.3 Å. This is fully overlapped at 2.2 µm sarcomere length, but at 2.5 µm sarcomere length the overlap is reduced by 1500 Å per half sarcomere to 5639.3 Å, so the overlapped actin filament length and the total attached head number would be expected to drop. The ratio 7139.3/5639.3 is 1.266, very close to what the density maps suggest (1.128 or 1.275). To match the 1.275 ratio value there would be 100% attachment, assuming full overlap also has 100% attachment. To match the 1.128 value (phase set 1, which we prefer) the attachment would be about 80%. However, if some of the lever arm mass has moved outside the 90 Å radius, as we suggest below, then this 80% will be an underestimate of the attachment number, which could well be significantly greater than 80%. We conclude that the numbers of myosin heads attached to a given length of the actin filaments are approximately the same in both cases, with probably much more than 80% attachment per unit actin filament length at 2.5 µm compared to full overlap rigor. Apparently it is not a significantly different attachment number per unit thin filament length that is making the two rigors look so different.

Note finally, that the fish sarcomere shows almost exact constant volume behaviour for the two lengths discussed here, the volumes being (3.29 ± 0.08) × 10^9^ Å^3^ at 2.2 µm and (3.27 ± 0.10) × 10^9^ Å^3^ at 2.5 µm. These are not significantly different (*p* = 0.85). The A(10) d-spacings change from 387 Å to 362 Å. The inter-myosin filament (*a* = *b*) spacing changes from 447 to 418 Å.

### 3.2. Analysis of the Actin Layer-Lines

#### 3.2.1. Steric Blocking by Tropomyosin 

The classic steric blocking mechanism of thin filament regulation by tropomyosin [26,27,28,29] had the tropomyosin strands in muscle thin filaments moving across the face of the underlying actin monomers when calcium binds to troponin, thus exposing the sites on actin to which the myosin heads could bind. This mechanism was determined using X-ray diffraction data from relaxed, active, and rigor muscles in which the main observation was an increase in intensity of the AL2 layer-line at a radius of around 0.02 Å^−1^ (arrowed as AL2 in Figure 4a). This radial position is exactly where the strong increase in the A2 from the short rigor muscle is seen (Figure 7b). The A2 peak can be seen clearly in Figure 4a. More recent work on thin filament regulation since 1972 has shown that much of the tropomyosin shift is a result of calcium ions binding to troponin when a muscle is activated, but that further tropomyosin movement can be produced by the binding of myosin heads in strong states (see Paul et al. [29] for recent discussion of this). It can be concluded that, in the full overlap rigor preparation with a large number of strongly bound rigor heads, the tropomyosin shift is probably as much as can occur and gives a strong AL2 peak.

Turning now to the longer sarcomere length rigor, the intensity increase of the AL2 is very much less than for the full overlap rigor. There is still a small increase compared with relaxed muscle, but the peak is at a slightly smaller reciprocal radius than for the full overlap rigor. What can be happening here? What is possible, since the number of attached heads per unit length of thin filament seems to be about the same as in the full rigor, is that the motor domains are not quite in the same position in the two rigors. If the tropomyosin position in the full overlap rigor has strongly attached heads that push tropomyosin to its fully on position, then perhaps the longer length rigor has motor domains which do not push the tropomyosin over so much.

#### 3.2.2. Analysis of the Higher Order Actin Layer Lines

Considering the rest of the low-angle actin layer-lines we look at the A6 first. This layer line at 1/59 Å^−1^ is one of the strongest actin layer lines and its increase in intensity in patterns from active and rigor muscles has been taken as an indication of myosin head attachment to actin. But in the longer length rigor the intensity increase occurs almost in the same radial position as the peak from resting muscle (Figure 7f and Figure 12a), whereas the full overlap rigor intensity has a very different profile with intensity tapering in towards the meridian. The conventional reason for seeing this shape in active and rigor muscle is that the myosin heads attached to actin are all following the helical symmetry of the actin filament. 

**Figure 11:**
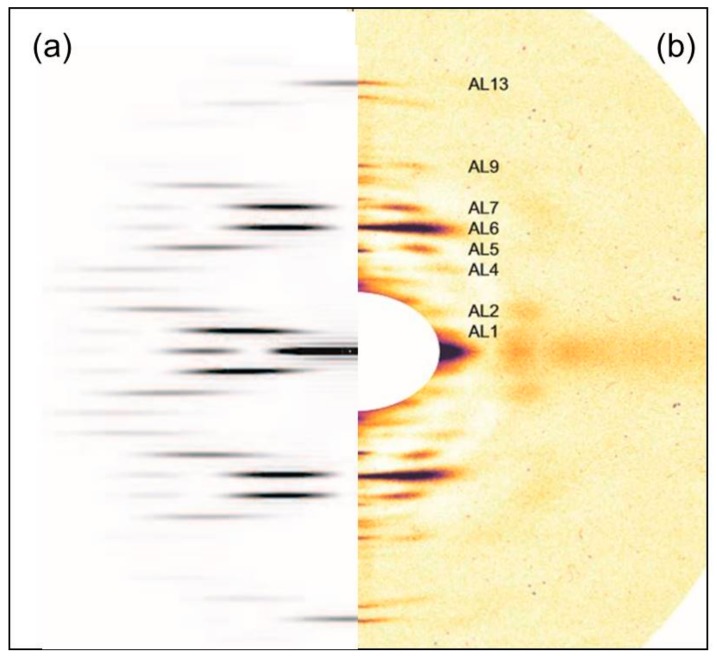
(**a**) The left half of a simulated diffraction pattern of a helix with the symmetry of the actin filament (a 13/6 helix of repeat 360 Å and subunit translation 27.36 Å) obtained with the program Helix [30]. Intensity from AL1 upwards gradually moves away from the meridian up to AL3 and then moves back towards the meridian up to AL6. Something similar then occurs from AL7 to AL13 which is on the meridian; (**b**) The right-hand half of part of the low-angle diffraction pattern from the full overlap rigor muscle (cf. Figure 2), with the same gernal shape of diffraction pattern, but with peaks closer to the meridian than is expected from just actin (**a**). For further details see text.

This means that not only are the motor domains attached to actin forming a partially occupied helix on actin with the same helical symmetry as the actin monomers, but the lever arm regions of the myosin heads are also following the same actin helical symmetry. The whole head is stereospecifically bound to actin as in Figure 1 and is actin-centred. All of the myosin head regions therefore contribute to the same layer lines as in Figure 11a. However, since the lever arms are at a much higher radius than the actin monomers (see Figure 1) their contribution to the AL6 layer line is further in towards the meridian than the original resting peak from actin and this adds the intensity taper towards the meridian seen on the AL6 in Figure 11b, and it gives peaks on the other actin layer lines such as AL4, AL5, and AL7 with intensity closer in to the meridian than an unlabelled actin filament would give, but showing the pattern of peaks and relative radial positions characteristic of something with the geometry of the actin helix (Figure 11). 

**Figure 12:**
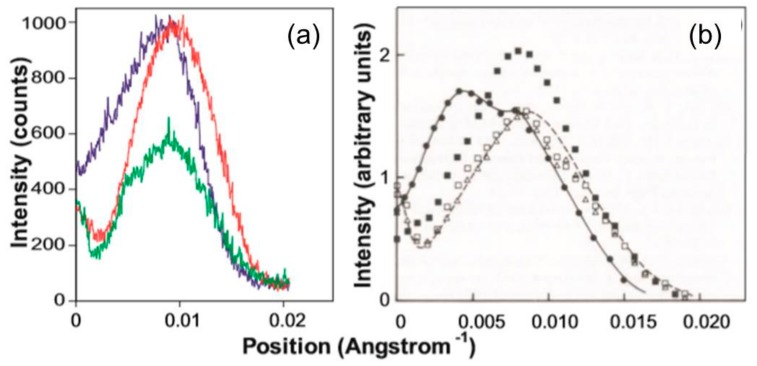
(**a**) Actin layer-line six intensity profiles from the 2.2 µm-rigor (blue), 2.5 µm-rigor (red), and relaxed (green) states from the laboratory X-ray data in this investigation; (**b**) Actin layer-line six intensity profiles from rest (open squares), active (black squares), and end of relaxation (open triangles) synchrotron X-ray patterns and rigor (black circles + solid line) and relaxed (dashed line) laboratory X-ray patterns from the work of Wakabayashi et al. [31], reproduced here with permission.

In summary, the inner part of the AL6 intensity from the 2.2 µm rigor muscles can be ascribed directly to the lever arms of the actin-attached myosin heads when they follow approximately the same helical symmetry as actin. This argument applies to both rigor and to a lesser extent active muscles (Figure 12). Note, however, that the regular rigor geometry, as in Figure 1, tilts the lever arms down and in towards the actin filament; it is a relatively compact structure. Why then does the intensity increase on the AL6 from the longer length rigor only occur at about the same radial position as the resting AL6 peak (Figure 7f)? The obvious conclusion is that, although the motor domains of the actin-attached myosin heads must be following the actin helical symmetry, the lever arms of these attached myosin heads cannot be so regularly organized; they do not follow the symmetry of the actin helix. One way in which this might happen is that the lever arms of the attached heads are somehow pointing back towards the myosin filaments from which they come. Another possibility is that the lever arms are just randomly disordered. However, we show later that the disorder would have to be of a special kind.

#### 3.2.3. Analysis of the Meridian

One of the characteristic features of the diffraction pattern from active and rigor vertebrate muscles is that the M3 reflection increases in spacing by 1% or more compared to the resting value of around 143 Å (143.2 Å for fish muscle [17]). This has been attributed to some sort of activation of the myosin filaments. There is apparently no calcium binding site on myosin so what this activation can be is not clear. Or it could just be that when the myosin heads all move away from the myosin filament surface in active or rigor muscle, the minimum energy position for the coiled-coils left in the filament backbone shifts to a new longer value than 143.2 Å; it may not need to be a specific activation process. We have seen above that the full overlap rigor and longer rigor muscles both show the same 1.54% increase in M3 spacing to an average of 145.7 Å. 

Figure 6a shows that the myosin meridionals change intensity on going from resting to the two rigors. But the changes are not the same for the two rigors. In particular, although the M6 remains about the same in all cases, the M9, M11, and M15 peaks all increase much more for the longer rigor muscles than the full overlap muscles. In addition the M3 (Figure 9 and Table 2) from the longer rigor is more than the short rigor intensity by a factor of around 2 (943.2/469.6 = 2.01; Table 2) and the M2 increases by about a factor of 1.4 in the longer rigor. Note also that the long rigor troponin peak is also larger than the full overlap rigor peak by a factor of 2.0. We do not know why this is. On the other hand, the M1/C-protein peak from the longer rigor is weaker than the short rigor peak by a factor around 3.2 (Table 2). 

Why should the meridional peaks be so different between the two rigors? It may be that the labelling pattern of heads on the actin filaments is different in the two cases, even though the attachment number per unit actin filament length seems to be about the same in both cases. However, it is not obvious why this should happen. Or it could be that the myosin heads attach to the same arrangement of actin monomers in each case, but the lever arms of the attached myosin heads are somehow marking the 145.7 Å periodicity better in the longer rigor structure. We know that rigor heads, even though they are attached to actin, can produce a stronger M3 peak because they are linked back to tethers on the myosin filament backbone. The labelling pattern itself can produce an enhanced M3 [8]. But what if the lever arms of these heads, which we know from above (the shape of AL6) are not following the actin helix, are oriented so that they are more or less perpendicular to the fibre axis? Then the main myosin meridional peaks would go up. But why do not M12, M13, and M14 go up too? This could simply be that they lie on the minimum of the transform of the lever arm mass at right angles to the fibre axis. M11, which increases, has a spacing of just under 40 Å. M12, M13, and M14 which stay the same, have spacings of 36.4, 33.6, and 31.2 Å, respectively and M15, which goes up, has a spacing of 29.1 Å. The minimum would therefore be between 1/29.1 and 1/40 Å^−1^, consistent with a projected mass onto the fibre axis of axial dimension around 30 to 40 Å. The axial thickness of the lever arms in Figure 1, when tilted back on the motor domain to project at 90° to the fibre axis is consistent with this. Such tilting would also take the myosin filament end of the lever arms further outside the 90 Å radius at which we were doing our mass calculations, reinforcing the idea that the attachment number per unit thin filament length is about the same in both rigors. 

## 4. Materials and Methods 

Handling and dissection of the flat fish (Plaice—*Pleuronectes platessa* and Turbot—*Psetta maxima*) fin muscles used in this study have been described in detail elsewhere [32,33]. Briefly, flatfish were obtained from London University Marine Biological Station (U.M.B.S.), Isle of Cumbrae, Scotland and from Aquarium Technologies Ltd., Weymouth, UK. The dissected muscles were mounted in Perspex X-ray chambers containing mylar windows at the front and back of the chamber to allow the passage of X-rays and laser light. There was a movable clamp to securely hold the fin bone and a slot at the top of the cell allowed an adjustable lever to enter the cell and hold the other end of the muscle. This could change the muscle length.

For the rigor muscle experiments, Plaice and Turbot fin muscles were chosen from close to the tail to be approximately 7 to 9 mm long. This was to optimise the preparation so that the muscles were as thin as possible to prevent ATP depletion in the core of the muscle, but still long enough to extend across the mylar window of the X-ray chamber. The fin muscles were dissected in cold modified Cobb Ringer (concentrations, mM: NaCl, 141; KCl, 5; MgCl_2_, 1; NaHCO_3_, 2; NaH_2_PO_4_, 2; Glucose, 5; pH 6.8). They were then pinned out onto a transparent silicon base, so that the fibres were not slack, and immersed in a beaker of fresh cold Ringer, constantly stirred by a magnetic flea.

Muscles were skinned by immersion in 5 mM ATP-relaxing solution containing the skinning agent TritonX-100 (concentrations mM: MgATP, 5; K_2_EGTA, 5; KProprionate, 150; MgAcetate, 4; Imidazole, 10; Triton-X 100, 1%) for four hours, then rinsed in ATP-relaxing solution minus Triton. Examination of the muscles under the light microscope was carried out to check that they were relaxed and not showing any signs of rigor, i.e., increased stiffness or opacity. The muscles were pinned out at the two different laser diffraction monitored sarcomere lengths (2.2 and 2.5 µm) and rigor was induced by replacing the relaxing solution by BDM-rigor solution (concentrations mM: K_2_EGTA, 2.5; KProprionate, 150, MgAcetate, 2; Imidazole, 10; BDM, 10). The muscles were soaked in this solution for at least 4 hrs to allow the BDM to diffuse through the whole muscle. BDM (2,3-butanedione monoxime) stops the muscles developing rigor force and hence reduces damage to the muscle entering rigor. The rigor muscles were mounted in the X-ray chamber with sufficient tension in the fibres to keep the preparation aligned across the mylar windows. The X-ray chambers were filled with rigor solution without BDM to record the X-ray diffraction patterns.

In the first study, X-ray diffraction patterns were taken using a laboratory based Elliot Big Wheel Rotating Anode generator, with a Huxley–Holmes Crystal Monochromator camera, and recorded on photographic film. Three kinds of X-ray diffraction pattern were recorded: one from relaxed muscles at SL = 2.45 µm, one from muscles put into rigor at the short SL of 2.2 µm, and one from muscles put into rigor at the longer SL of 2.5 µm. A total exposure time of 8 h was obtained for each of the three patterns. Recorded patterns on film were digitally scanned using a LeafScan scanner at a resolution of 10 µm per pixel. Using the program FibreFix [18], the diffraction patterns were centred and aligned with their meridian vertical and equator horizontal. Background scatter in the patterns was removed using the FibreFix 2D smooth background fitting algorithm. Quadrant folding was carried out on the three patterns to average the intensity in the four quadrants of the patterns. The total intensity of each pattern was made the same so that the three patterns could be compared and difference maps were taken. The central portion of these X-ray diffraction patterns saturated the film on which they were recorded, therefore, this section was masked out of all the patterns and difference maps.

In the second study, X-ray diffraction patterns were recorded on beamline ID2 bypass at the ESRF Grenoble using the RAPID detector [34]. X-ray patterns were taken from eighteen muscles put into rigor at the two different sarcomere lengths, with a maximum exposure of 11 s for each muscle. This gave a total exposure, after summation of the patterns, of 82.0 s for SL = 2.2 µm and 90.5 s for SL = 2.5 µm. Patterns were selected that were reasonably straight with respect to the attenuation strip along the equator so that none of the equatorial reflections were lost off the edge of the strip. Using the program FibreFix [18], the chosen patterns were corrected for camera background scattering by removing the X-ray pattern taken from the apparatus without a muscle present. The patterns were centred and aligned with their meridian vertical and equator horizontal so that the patterns at each sarcomere length could all be added together to produce two summed patterns, one at SL = 2.2 µm and one at SL = 2.5 µm. Quadrant folding was carried out on the summed patterns. Background scatter in the summed patterns was removed using first the 2D roving window fitting algorithm and then the 2D circularly symmetric fitting algorithm in FibreFix. Meridional and equatorial intensity profiles were obtained using the HOR and VER tools in FibreFix and peaks were fitted using the Lorentzian area tool in Peakfit (Available online: https://systatsoftware.com/products/peakfit/).

## 5. Conclusions

**Figure 13:**
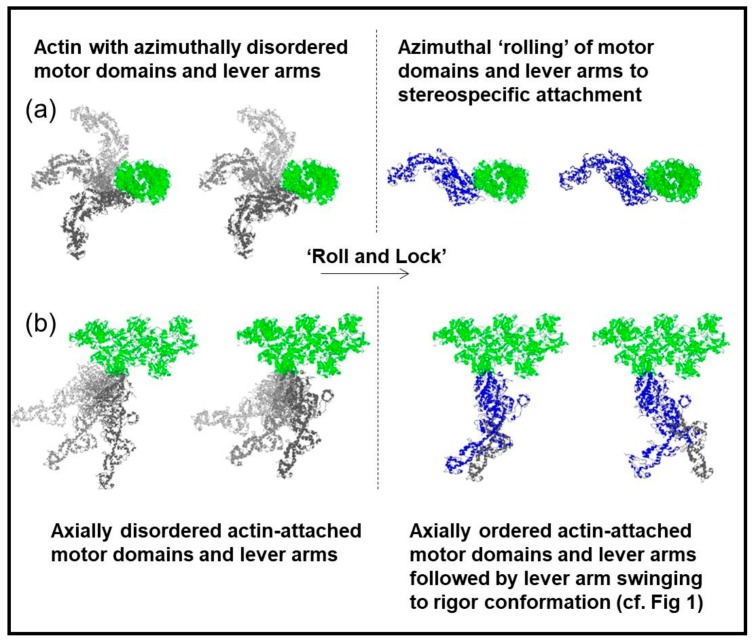
Distinct attached states of myosin heads (blue or grey) bound to actin (green) with the actin filament axis end on (**a**) or in the plane of the page (**b**). In (**a**) left the myosin heads are azimuthally disordered, whereas in (**a**) right there is sterospecific binding of the heads to actin so that they follow the actin helical symmetry. In (**b**) left the heads are axially disordered whereas in (**b**) right they have become stereospecifically attached to actin and axial lever arm swinging can occur. Adapted from Figure 6 of Ferenczi et al. [35], and reproduced with permission.

To summarise our results on the two rigors, we have found evidence that fish muscles put into rigor at a sarcomere length of 2.2 µm have the myosin heads attached to actin in the typical rigor conformation illustrated in Figure 1. But, if the muscle is put into rigor at a sarcomere length of 2.5 µm, the myosin head configuration is different. There appear to be roughly the same number of heads attached for a given length of actin filament, but at 2.5 µm the motor domains appear to be attached differently because the tropomyosin does not shift as much as at full overlap. The myosin lever arms appear azimuthally disordered because the AL6 layer line does not have the increased intensity towards the meridian characteristic of the lever arms conforming to the symmetry of the actin helical symmetry, and the meridional reflections suggest that the lever arms are much more perpendicular to the fibre axis than in the full rigor. These results are very reminiscent of the ‘Roll and Lock’ mechanism of Ferenczi et al. [35], where they suggested that force production in the myosin cross-bridge cycle on actin was a two-step process. The transition from weak to strong binding would be the first step, a ‘roll and lock’ mechanism, and the second step would be the tilting of the lever arm on the steroespecifically bound motor domains. We have adapted part of their Figure 6 as our Figure 13. Here it shows that heads might bind to actin initially with their motor domains and lever arms azimuthally and axially disordered, after which there is a transition to attached states where the motor domains attach stereospecifically to actin. After this stereospecific attachment, the lever arms could then swing on actin towards the rigor conformation as required for force generation and muscle shortening. Our results from the longer sarcomere length rigor suggest conformations similar to Figure 13 left half except that the lever arms in (b) would generally be roughly perpendicular to the fibre axis. The structure in the full overlap rigor would be as in Figure 13 right except that the lever arms would be in the ‘swung’ position (bottom right) as in Figure 1.

This is a surprising result and we question why it should happen. One possibility is that in muscles set to 2.5 µm and held there, the myosin and actin filaments might have much less freedom of axial movement than at the shorter sarcomere length and the initial attachment state of the heads (non-stereospecific labelling of actin) may for some reason be locked in; they cannot go through the ‘roll and lock’ stage to full rigor. Have we inadvertently trapped the initial binding state in active muscle of myosin heads on actin? 

We do not yet know if something similar happens in other muscles, or how bony fish muscle behaves in rigor at other sarcomere lengths than the ones we have chosen. Also beyond the scope of the present paper is any analysis of the relative mechanical properties of the two rigors. There is much more to be done on this interesting new state.

## Figures and Tables

**Table 1:** Average intensities of the first five equatorial X-ray reflections from Plaice fin muscle put into rigor at SL = 2.2 and 2.5 µm. In both cases the mean A(10) intensity was set at 100 and the number of measurements was nine. The numbers quoted here are based on the total number of counts in each peak from the nine muscles in each case. Uncertainties are difficult to estimate (<1% from counting statistics, but perhaps up to 5% between muscles).

Reflection	Average Observed Intensity for Rigor at 2.2 µm	Average Observed Intensity for Rigor at 2.5 µm
A(10)	100	100
A(11)	231.2	104.8
A(20)	18.4	12.4
A(21)	19.6	14.7
A(30)	6.6	0.86

**Table 2:** Measured values for the intensity, spacing, and cross-meridional widths of the four main meridional peaks visible in the two summed X-ray patterns from Plaice muscles put into rigor at long and short sarcomere length. The spacings were calibrated using the measured position of the M3 reflection above and the peaks were fitted using the program Peakfit. The peak intensities were corrected for width differences in the peaks by multiplying the measured intensity by the FWHM of each peak measured perpendicular to the meridian [19]. See Appendix A for details and justification of the width correction.

Measured Parameter	M1/C-Protein		Troponin		M2		M3	
	2.2 µm	2.5 µm	2.2 µm	2.5 µm	2.2 µm	2.5 µm	2.2 µm	2.5 µm
Intensity (×10^5^ counts)	4.581	0.917	2.187	3.588	5.519	5.226	5.018	6.999
Spacing (×10^−3^ Å^−1^)	2.248	2.254	2.579	2.572	4.469	4.514	6.865	6.865
FWHM (×10^−4^ Å^−1^)	3.08	4.80	4.18	5.42	5.42	8.04	9.36	13.48
Corrected Intensity	141.34	43.94	91.26	194.5	299.8	420.2	469.6	943.2

**Figure A1:**
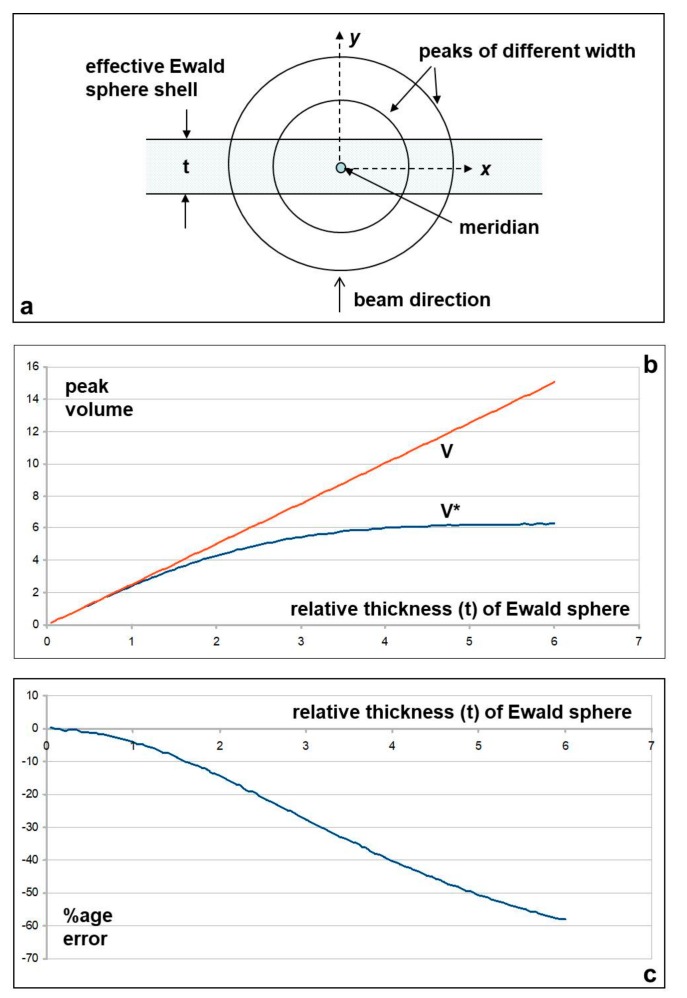
(**a**) Geometry of the diffraction pattern looking down the meridian. The two circles represent the sizes of two meridional peaks of different width across the meridian. The horizontal lines represent the thickness of the Ewald sphere relating to an X-ray beam where the wavelength spread can be represented by a thickness *t*; (**b**) Graphs showing the true volume *V* and the estimated volume V* as a function of Ewald sphere thickness *t*; (**c**) Graph showing the %age error relative to Ewald sphere thickness t, of multiplying the meridional peak area by the full width at half maximum of the peaks (see Table 2).

## Abbreviations

ATP	Adenosine triphosphate
BDM	3,2-Butanedione monoxime
IFM	Insect flight muscle
SL	Sarcomere length
EGTA	Ethylene glycol-bis(β-aminoethyl ether)-*N*,*N*,*N*’,*N*’-tetraacetic acid

## Appendix A. Correcting the M3 and Other Meridional Intensities

We have checked the intensity correction that is usually applied to the M3 (Huxley et al., 1982 [19]) by a full estimation of the errors involved. The conventional correction is to multiply the observed intensity of the M3 peak by its full width at half maximum. We call the full width W. The present analysis assumes that the M3 and other meridional peaks are approximately Gaussian in profile: *G*(*x*,*y*) = *A* exp[−(*x*^2^ + *y*^2^)/2σ^2^], where *x* and *y* spread in orthogonal directions perpendicular to the meridian of the diffraction pattern (Figure A1a). The full volume of this peak is *V* = 2πσ^2^. The observed intensities are the volumes in these peaks as sampled by the Ewald sphere of radius 1/λ where λ is the wavelength of the X-ray beam. The complication is that the Ewald sphere is not infinitely thin; we assign to it an effective thickness *t*.

The conventional estimation of volume (*V**) is the observed intensity *I*, which is the cross-sectional area of the Gaussian (*A* = σ√(2π)) multiplied by the effective thickness of the Ewald sphere (*t*) as long as *t* is small. From Figure A1a it can be seen that the intensity profile parallel to the plane of the Ewald sphere becomes less and less like the central section as one moves away from this section out towards the Ewald sphere edges. That is why *t* must be small for the correction to work. To get the estimated full volume of the peak, *I* is then multiplied by the Gaussian width at half height (*W* = 2σ√(2ln2)). So *V** = *A* × *t* × *W* = 4tσ^2^√(π ln2) if *t* is small.

For two peaks of different widths σ_1_ and σ_2_ the estimated volume ratio is:

*V**_1_/*V**_2_ = 4tσ_1_^2^√(π ln2)/4tσ_2_^2^√(π ln2) = σ_1_^2^/σ_2_^2^ as long as *t* is small.

From above, the correct volume ratio is *V*_1_/*V*_2_ = 2πσ_1_^2^/2πσ_2_^2^ = σ_1_^2^/σ_2_^2^ which is exactly the same as *V**_1_/*V**_2_. So the usual estimation is good as long as *t* is small.

We can then estimate the levels of error involved for peaks of different widths relative to the Ewald sphere thickness *t*. The beams at modern synchrotrons such as the ESRF or Argonne typically have energy spreads (hence λ spreads) in the X-ray beams through low-angle beam-lines of about Δ*E*/*E* = 2 × 10^−4^. This spread in the Ewald sphere width will itself be something like Gaussian in profile, but we have approximated the Ewald sphere to being a uniform shell which has thickness *t*. We have also simplified the calculation by assuming throughout that *A* = σ = 1.

The question to answer is how well does the observed intensity peak multiplied by the width at half height W (i.e., the volume *V**) represent the true volume (*V*) of the diffraction peak. We have carried out these calculations by numerical integration using Monte Carlo methods and the results are plotted in Figure A1b,c. These show that for t values which are less than about 0.5, the percentage error in using the usual method of intensity correction is less than 1%. From exact calculation the width W of the Gaussian is *W* = 2σ√(2ln2)~2.35σ; in our case σ = 1 so *W* = 2.35. Therefore, the error in the intensity estimation is less than 1% when *t*/*W* is less than 0.5/2.35 = 0.2.

Estimated widths *W* from our own and previously published data on the muscle M3 from synchrotron beamlines with a small focus (e.g., ESRF, Argonne) are of the order of 0.001 Å^−1^ for the M3 in patterns from resting muscle and about 0.002 Å^−1^ in patterns from active muscle. Since the Ewald sphere thickness (*t*) is of the order of 0.0002 Å^−1^ (i.e., a spread of 2 × 10^−4^ in an Ewald sphere of radius ~1 Å^−1^) the experimental values of *t*/*W* that we are dealing with are about 0.2 for relaxed muscle and 0.1 for active muscle. These are about equal to (relaxed muscle) or better than (active muscle) our 1% error limit. Looking at the rigor widths in Table 2, the full widths at half maximum (FWHM) are all below 0.001 except for the M3 at the longer rigor which is 0.00135. All of these are within the limit for 1% accuracy. We conclude that the correction that is usually applied to the M3 intensity and other meridionals is valid to within about 1% and that there is, therefore, a real increase in M3 intensity on activation or going into rigor.
